# Hypoxic Conditions Promote Rhythmic Contractile Oscillations Mediated by Voltage-Gated Sodium Channels Activation in Human Arteries

**DOI:** 10.3390/ijms22052570

**Published:** 2021-03-04

**Authors:** Anne Virsolvy, Aurélie Fort, Lucie Erceau, Azzouz Charrabi, Maurice Hayot, Franck Aimond, Sylvain Richard

**Affiliations:** 1 PhyMedExp, Université de Montpellier, INSERM, CNRS, 34495 Montpellier, France; aurelie.fort@inserm.fr (A.F.); lucie.erceau@hotmail.fr (L.E.); azzouz.charrabi@inserm.fr (A.C.); m-hayot@chu-montpellier.fr (M.H.); franck.aimond@inserm.fr (F.A.); sylvain.richard@inserm.fr (S.R.); 2 CHU de Montpellier, 34495 Montpellier, France

**Keywords:** human arteries, smooth muscle, sodium channels, hypoxia, vasomotion

## Abstract

Arterial smooth muscle exhibits rhythmic oscillatory contractions called vasomotion and believed to be a protective mechanism against tissue hypoperfusion or hypoxia. Oscillations of vascular tone depend on voltage and follow oscillations of the membrane potential. Voltage-gated sodium channels (Na_v_), responsible for the initiation and propagation of action potentials in excitable cells, have also been evidenced both in animal and human vascular smooth muscle cells (SMCs). For example, they contribute to arterial contraction in rats, but their physiopathological relevance has not been established in human vessels. In the present study, we investigated the functional role of Na_v_ in the human artery. Experiments were performed on human uterine arteries obtained after hysterectomy and on SMCs dissociated from these arteries. In SMCs, we recorded a tetrodotoxin (TTX)-sensitive and fast inactivating voltage-dependent I_Na_ current. Various Na_v_ genes, encoding α-subunit isoforms sensitive (Na_v_ 1.2; 1.3; 1.7) and resistant (Na_v_ 1.5) to TTX, were detected both in arterial tissue and in SMCs. Na_v_ channels immunostaining showed uniform distribution in SMCs and endothelial cells. On arterial tissue, we recorded variations of isometric tension, ex vivo, in response to various agonists and antagonists. In arterial rings placed under hypoxic conditions, the depolarizing agent KCl and veratridine, a specific Na_v_ channels agonist, both induced a sustained contraction overlaid with rhythmic oscillations of tension. After suppression of sympathetic control either by blocking the release of catecholamine or by antagonizing the target adrenergic response, rhythmic activity persisted while the sustained contraction was abolished. This rhythmic activity of the arteries was suppressed by TTX but, in contrast, only attenuated by antagonists of calcium channels, Na^+^/Ca^2+^ exchanger, Na^+^/K^+^-ATPase and the cardiac Na_v_ channel. These results highlight the role of Na_v_ as a novel key element in the vasomotion of human arteries. Hypoxia promotes activation of Na_v_ channels involved in the initiation of rhythmic oscillatory contractile activity.

## 1. Introduction

Vasomotion is a mechanism of physiological importance inherent to the smooth muscle wall observed both in vivo and in vitro. It consists of rhythmic oscillations in vascular tone of blood vessels, which leads to flow motion corresponding to periodical blood flow fluctuations [1]. Vasomotion is described as a compensatory mechanism for enhancing tissue oxygenation in conditions of reduced oxygen supply, suggesting a protective role in situations where perfusion is critically limited [2,3]. Its influence is modified under pathological conditions and can be enhanced under ischemic or hypoxic conditions [4,5,6]. Originating in the vessel wall and with low frequencies [7], oscillations of resting tone are associated with parallel and synchronized oscillations both in membrane potential and in intracellular Ca^2+^ concentration in SMCs [5,8]. The mechanism of vasomotion generation is thought to be a voltage-dependent coupled model [1] involving depolarization and calcium (Ca^2+^) influx through voltage-gated Ca^2+^ channels as essential steps in coordinating the individual oscillators [9]. Rhythmic oscillations in membrane potential may be recorded in SMCs [10,11,12] although these cells belong to the category of non-excitable cells.

Voltage-gated sodium channels (Na_v_) are molecular characteristics of excitable cells. They are responsible for the initiation and propagation of action potential [13]. Although these channels support electrogenesis, they are expressed and play functional roles in cell types that are not considered electrically excitable like SMCs [14]. Na_v_ channels are present in SMCs where voltage-gated Na^+^ currents (I_Na_) have been recorded in various mammalian and human cell types [15,16,17,18,19,20]. The implication of tetrodotoxin (TTX)-sensitive Na_v_ channels in arterial contraction has been established in animal models [21,22]. These channels are involved at the sympathetic perivascular nerve terminals level through release of catecholamine and α1-adrenergic receptors pathway and in SMCs via a regulation pathway that involves Ca^2+^ influx through voltage activated Ca^2+^ channels and Na^+^/Ca^2+^ exchanger (NCX) activated by Na^+^ influx consecutive to depolarization-induced Na_v_ channels activation [21]. To date, there is no information regarding a possible role of Na_v_ channels in the regulation of human arterial contractile activity as in vasomotion despite the voltage-dependence of this phenomenon.

In the present study, we investigated the functional role of Na_v_ channels in human uterine artery. We determined the expression profile of Na_v_ genes and we established conditions that allowed us to specifically target SMCs Na_v_ channels and couple their activity to vasoconstriction. Our results indicated that under physiopathological hypoxic condition, Na_v_ channels might initiate and maintain vasomotion.

## 2. Results

### 2.1. Spontaneous Contractile Oscillations

**Figure 1:**
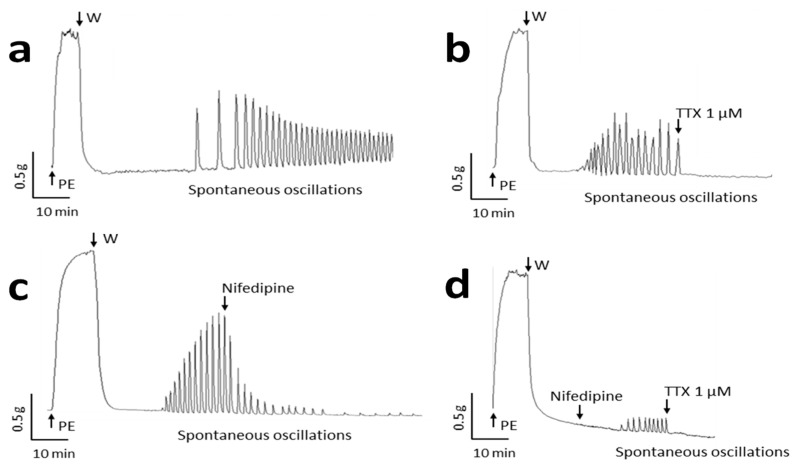
Spontaneous contractile activity occurring in human uterine artery is sensitive to tetrodotoxin (TTX). Panels show original recordings of isometric tension variations in human arterial rings illustrating (**a**) spontaneous contractile oscillations (*n* = 15); (**b**) the effects of 1 µM TTX (*n* = 10) and (**c**) 1 µM nifedipine (*n* = 5) on spontaneous oscillations and (**d**) spontaneous oscillations occurring in the presence of 1 µM nifedipine and abolished by subsequent addition of 1 µM TTX (*n* = 5). Phenylephrine 10 µM (PE) was first added to assess arterial ring contractility then washed (W).

Spontaneous contractile oscillations were evidenced in several uterine arterial rings (Figure 1a). This rhythmic activity appeared unpredictable and not specifically inducible. Oscillations occurred in arterial segments with an incidence lower than 20% (corresponding to 15 of the 80 specimens used in the study), a mean frequency of 3.2 ± 0.4 min^−1^ and mean amplitude corresponding to 29.2 ± 8.1% of phenylephrine (PE)-induced maximal contraction. Tetrodotoxin (TTX, 1 µM), a highly specific Na_v_ channel blocker, inhibited fully this spontaneous contractile activity (Figure 1b). The oscillations not only ceased instantly after the addition of the toxin but also never initiated in its presence in arterial segments expected to exhibit such activity. The L-Type Ca^2+^ channel blocker, nifedipine (1 µM), partially inhibited spontaneous contractile activity with a progressive slow decline of the amplitude and a slight decrease in the frequency of oscillations (2.18 ± 0.33 min^−1^) (Figure 1c). After 20 min, the amplitude of oscillations was reduced by 71.1 ± 2.8% when compared to their amplitude before addition of nifedipine (*n* = 5). Furthermore, in the presence of nifedipine, oscillations could still initiate, but with lower amplitude (4.75 ± 1.07% of PE-induced contraction) and frequency (2.26 ± 0.35 min^−1^, *n* = 5) (Figure 1d). They were stopped by subsequent addition of TTX. This remarkable effect of TTX prompted us to envisage the presence of Na_v_ channels in human arteries and their functional role in vasomotion.

### 2.2. Electrophysiological Characterization of I_Na_ and Identification of Na_v_ Channels in Uterine Artery SMCs

**Figure 2:**
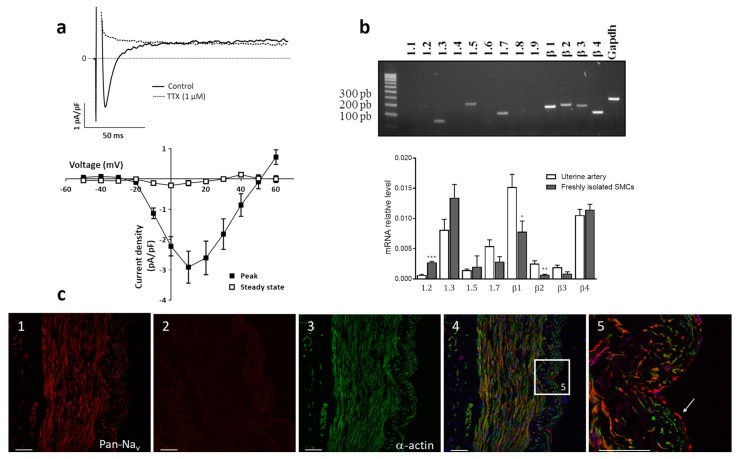
(**a**) Upper panel: Representative I_Na_ current traces recorded at +10 mV, on SMCs isolated from human uterine artery in control (plain line) and after 1 µM TTX (dotted line). Lower panel: Mean ± s.e.m TTX-sensitive I_Na_ current/voltage relationships measured at the peak current and at steady-state (*n* = 11). (**b**) Analysis of Na_v_ transcripts in total RNA extracted from human uterine artery and freshly isolated SMCs. The upper panel illustrates representative amplification obtained after end-point RT-PCR performed on uterine artery. GAPDH primers were used as positive controls to validate reverse transcription. The graph shows evaluation performed by quantitative real-time RT-PCR for Na_v_ isoform transcripts with Ct < 40. Transcript levels were normalized to that of the GAPDH housekeeping gene in each sample and compared in the whole arterial tissue, and freshly isolated SMCs. Experiments were performed in triplicate, *n* = 5. * *p* < 0.05; ** *p* < 0.01; and *** *p* < 0.001, comparing uterine artery whole tissue and freshly isolated SMCs for each gene with an unpaired *t*-test. (**c**) Immunolocalization of Na_v_ channels in uterine artery. Confocal microscopy images show typical labeling of uterine artery section with Pan Na_v_ channel antibody (**1**) and in the presence of peptide antigen negative control (**2**) (SP19 red fluorescence); α-actin antibody (green fluorescence) (**3**); merged fluorescence with TOTO counterstaining (blue) (**4**) and using a ×20 objective. (**5**) corresponded to high magnification (60× objective) of the inset in (4) showing endothelial (plain arrow) and smooth muscle (dotted arrow) cells. Scale bars: 50 µm. Enlarged images are displayed as supplemental data.

Using the whole-cell configuration of the patch-clamp technique, we investigated the presence of functional Na_v_ channels in human uterine artery SMCs. We evidenced an I_Na_ current blocked by 1 µM TTX (Figure 2a). Peak amplitude of this TTX-sensitive I_Na_ current was −2.91 ± 0.53 pA/pF at +10 mV characterized by a fast inactivating component (τ_inac_ = 6.8 ± 1.0 ms).

The molecular identification, determined by RT-PCR, revealed several isoforms of Na_v_ channels both in arterial tissue and in isolated SMCs (Figure 2b). Four transcripts of alpha-subunits were detected: Na_v_ 1.2, Na_v_ 1.3, Na_v_1.5 and Na_v_1.7. All beta-isoforms were present in total tissue and in freshly dissociated SMCs. Na_v_ 1.3 and Na_v_ 1.7 seemed to be most consistently detected. However, Na_v_ 1.5 and Na_v_ 1.2 were also present with the latter being more expressed in SMCs.

To determine the cellular localization of Na_v_ channels, arterial tissue sections were double-labeled with an anti-Pan antibody (red fluorescence) and a smooth muscle α-actin antibody (green fluorescence) (Figure 2c). Red fluorescence was uniformly observed throughout the media, intima and endothelium reflecting the presence of Na_v_ channels distributed in the endothelium and in the media layer (Figure 2c panel 1). They located in SMCs identified by green labeling for smooth muscle α-actin (Figure 2c panels 2 and 3) and in endothelial cells (Figure 2c panel 4). Thus, electrically active Na_v_ channels are present in arterial tissue and in SMCs of the uterine artery and they could be functionally coupled to arterial contraction.

### 2.3. Na_v_ Channels Activation Induced Vascoconstriction

**Figure 3:**
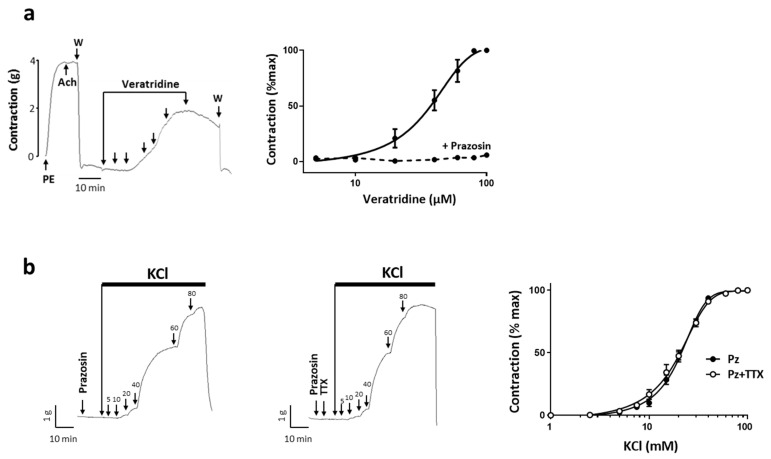
(**a**) Veratridine-induced vasoconstriction. Right panel shows typical recordings of the isometric response to cumulative doses of veratridine. The integrity of the arterial ring was first evaluated by the response to a sub maximal dose of phenylephrine (PE; 10 µM). Then the absence of endothelium was assessed by the absence of relaxation induced by Ach (1 µM). After washing (W), increasing concentrations of veratridine, from 5 to 100 µM, were added (arrows). Left panel represents concentration–response curves of veratridine in the absence and in the presence of prazosin 50 µM preincubated for 15 min before addition of veratridine (dotted line in the right panel showing averaged data). (**b**) Effect of TTX on the contraction induced by KCl. Upper panels show representative isometric tension variations induced by cumulative addition of KCl concentrations after a 10 min preincubation period with prazosin (50 µM) in the absence or in presence of TTX (1 µM). The graph represents dose–response curves for KCl in the presence of prazosin (●) and in the presence of prazosin plus TTX (○). Values are means ± s.e.m. of tension normalized to maximal KCl response. For each protocol, experiments were performed in duplicate, *n* = 15.

To investigate the functional role of these channels in the contractile responses, we used experimental protocols that we had previously established to unmask Na_v_ channels activity in rat aorta [21]. As Na_v_ channels seemed to localize also in endothelial cells (Figure 2c panel 4), all experiments were performed on arterial rings without endothelium to specifically target Na_v_ channels in SMCs, which was validated by a lack of response to Ach. First, we observed that veratridine, a Na_v_ channel agonist, induced a tonic and dose-dependent contraction of uterine artery (Figure 3a) with an EC_50_ value of 33.8 ± 2.3 µM and a maximal contractile response reaching 59 ± 15% of Phe-induced contraction. In the presence of prazosin (10 µM), an α1-adrenergic receptor antagonist, the effect of veratridine on contraction was suppressed (Figure 3a). This confirmed that the veratridine response was primarily mediated through activation of Na_v_ channels at perivascular nerve endings level via catecholamine release as we described in rat aorta [21]. To target specifically SMCs Na_v_ channels, we studied the effect of TTX on the KCl-induced contractile response in the presence of prazosin. We observed that 1 µM TTX had no effect on the KCl response whatever the concentration (Figure 3b). We found no modification of either KCl sensitivity (EC_50_), maximal-induced contraction (E_max_) or the response induced by 20 mM KCl-induced (E_20_) in the presence of TTX (Table 1). Under these experimental conditions, Na_v_ channels could not be activated by KCl-induced depolarization. We therefore hypothesized that this could reflect a depolarized membrane potential inducing inactivation of Na_v_ channels thereby unavailable for opening under basic physiological condition.

### 2.4. Hypoxia Unmasked the Participation of the SMCs Na_v_ Channel to Contraction

**Figure 4:**
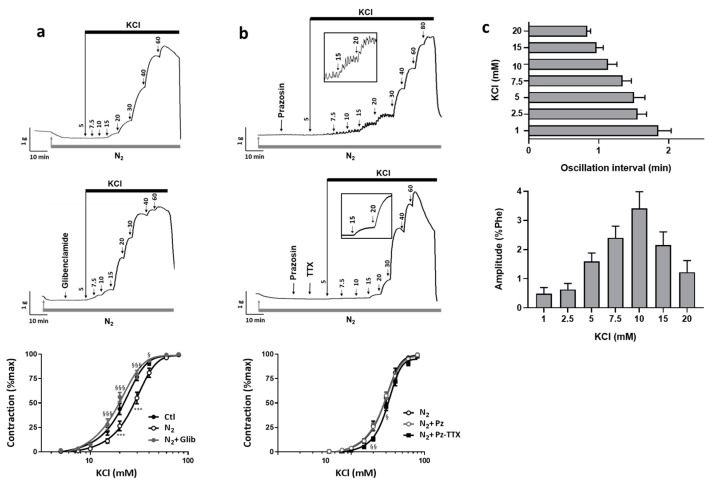
Effect of hypoxia on the contractile response to KCl and involvement of Na_v_ channels. The contractile responses to cumulative concentrations of KCl were characterized under hypoxic condition in the absence and in the presence of various antagonists: glibenclamide, prazosin and TTX. Hypoxic condition was reached by substitution of O_2_ by N_2_ bubbling in the organ bath. Upper panels show representative recordings of the variations of isometric tension according to the following experimental protocols: (**a**) in the absence (*n* = 20) and in the presence of glibenclamide (100 nM) (*n* = 10), (**b**) in the presence of prazosin (10 µM) and prazosin plus TTX (1 µM) (*n* = 15). Graph represents dose–response curves for KCl under each condition. Data representing means ± s.e.m. of tension normalized to maximal KCl response were analyzed with a non-linear fit function to determine EC_50_ values. For each protocol, experiments were performed in duplicate. Two-way ANOVA followed by the Bonferroni post-hoc test was performed for statistical analysis. * and § compare values respectively vs. CTL and vs. N_2_. § *p* < 0.05; §§ *p* < 0.01; *** and §§§ *p* < 0.001. (**c**) Bar graphs represented quantification of oscillations, which developed during KCl responses with the average interval between two oscillations (upper) and the average amplitude (lower). Values are mean ± s.e.m (*n* = 6).

Hypoxia induces hyperpolarization of vascular tissue in part through K_ATP_ channels activation. We reassessed the KCl-induced contractile response of uterine artery under hypoxic conditions. To induce hypoxia, we substituted oxygen (O_2_) by nitrogen (N_2_) bubbling in the organ chamber. After less than 5 min, O_2_ level in the organ bath medium drastically fell by 80% and stabilized within 10 min. In that hypoxic condition, the response to KCl was modified. We observed decreases in contractility and sensitivity to KCl as depicted by the E_max_ value and the rightward shift of the KCl dose–response curve with an increase of the EC_50_ value (29.1 ± 2.1 mM vs. 22 ± 1.3 mM under basal O_2_ condition) (Figure 4a for illustration, and Table 1). In the presence of glibenclamide (100 nM) a K_ATP_ channel blocker, although maximal contractile response was still reduced, the change in KCl sensitivity induced by hypoxia was suppressed (EC_50_ = 20.2 ± 1.3 mM, not different from basal O_2_ condition) (Figure 4a). Altogether, our results were in line with hypoxia-induced hyperpolarization of arterial tissue consecutive to K_ATP_ channel activation.

Thus, to unmask a possible involvement of Na_v_ channels in the KCl-induced contractile response, we evaluated under hypoxic condition and in the presence of prazosin (10 µM) the effect of TTX. The TTX (1 µM) inhibited the contractile response to KCl (Figure 4b, N_2_-Pz-TTX) for concentrations below 30 mM. For 20 mM KCl, 50% of the contractile response was suppressed by TTX (12.0 ± 1.8% vs. 26.4 ± 3.3% of max, *p* = 0.0256) (Table 1). The EC_50_ value for the KCl dose–response in the presence of TTX tended to increase when compared to the KCl response in absence of TTX (32.7 ± 1.3 vs. 29.3 ± 1.4 mM, *p* = 0.067) (Table 1). Additionally, in several arterial rings, an oscillatory activity superimposed to the tonic contraction for these KCl concentrations below 30 mM (Figure 4b, inset of the left panel). When observed, this oscillatory activity occurred between 2.5 and 20 mM KCl with concentration-dependent changes of frequency and amplitude. Frequency followed of the increase in KCl concentration, while amplitude followed a bell-shaped curve with a maximum for 10 mM KCl representing 3.4 ± 0.6% of Phe-induced contraction (Figure 4c). For KCl concentrations higher than 20 mM, oscillations ceased and were no more observed, this might reflect inactivation of Na_v_ channels unavailable for opening due to stronger depolarization. These oscillations occurred in 6 of the 15 vessel specimen submitted to that protocol (40%). It should be noted that such oscillations were never recorded in a non-hypoxic condition, or in the presence of TTX or of glibenclamide under a hypoxic condition, regardless of the KCl concentration. Similarly, the spontaneous oscillatory activity shown in Figure 1 was never detected under the hypoxic condition.

### 2.5. Na_v_ Channels Activation Triggers Vasomotion under the Hypoxic Condition

**Figure 5:**
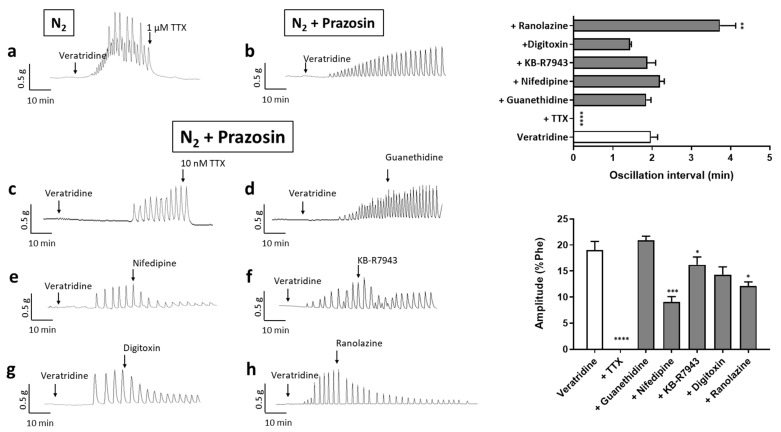
Characterization of veratridine-induced vasomotion under hypoxic conditions. All panels show representative recordings of isometric tension in human arterial rings and illustrate the response to veratridine (100 µM) under hypoxic condition (*n* = 5) (**a**) and the effects of various antagonists on the veratridine-induced vasomotion: (**b**) prazosin (10 µM); (**c**) TTX (10 nM) (*n* = 10); (**d**) guanethidine (5 µM) (*n* = 5); (**e**) nifedipine (1 µM) (*n* = 5); (**f**) KB-R7943 (10 µM) (*n* = 4); (**g**) digitoxin (10 µM) (*n* = 4) and (**h**) ranolazine (10 µM) (*n* = 5). Graphs summarize the interval between two oscillations and amplitude of oscillations averaged over the 20 min following the addition of the drug and in the presence of prazosin. Values are mean ± sem. Statistical analysis, comparing oscillation parameters before and after drug addition, was performed with a paired *t*-test for each condition. * *p* < 0.05; ** *p* < 0.01; *** *p* < 0.001; **** *p* < 0.0001.

To confirm the involvement of Na_v_ channels in the oscillatory activity, we evaluated the effects of veratridine under the hypoxic condition. When O_2_ was replaced by N_2_ in the organ bath, veratridine (100 µM) induced a sustained contraction, which was superimposed by oscillatory activity (Figure 5a). Both phenomena were suppressed after the addition of TTX (1 µM) with the return of arterial tone to the basal level (Figure 5a). The sustained contraction represented 44.3 ± 14.0% of Phe-induced response (Figure S2). Oscillations frequency and amplitude were 0.78 ± 0.24 min^−1^ and 24.2 ± 5.3% of Phe-induced contraction, respectively (*n* = 5). In the presence of prazosin (10 µM), only the oscillatory activity occurred (Figure 5b). Rhythmic contractile oscillations, observed in all the vessels treated, initiated between 2 and 40 min after addition of veratridine in the organ bath, in average around 15 ± 8 min, with a mean frequency of 1.97 ± 0.17 min^−1^ and a mean amplitude corresponding to 19.1 ± 2.7% of Phe-induced maximal response. Oscillations ceased immediately upon TTX addition, even for concentrations as low as 10 nM (Figure 5c) and were reinducible (not shown). Guanethidine (5 µM), an inhibitor of catecholamine release, had no effect on oscillatory activity (Figure 5d). Nifedipine (1 µM), a calcium antagonist, attenuated but did not suppress the oscillations of arterial tone (Figure 5e). A progressive decrease in amplitude was observed with a lower mean value but no significant incidence on frequency (Figure 5e). The residual activity was stopped by subsequent addition of 1 µM TTX. Slight effects, similar to that of nifedipine, were observed with KB-R7943 (Figure 5f) and digitoxin (*p* = 0.0564 for amplitude) (Figure 5g) suggesting partial and non-essential involvement of respectively the Na^+^/Ca^2+^ exchanger and the Na^+^/K^+^-ATPase. Otherwise, ranolazine, an inhibitor of cardiac Na_v_ channels, modified the oscillations (Figure 5h). The amplitude progressively decreased and the interval between two oscillations increased. Thus, amplitude and frequency were both significantly reduced.

## 3. Discussion

In the present study, we identified Na_v_ channels in human uterine artery and describe for the first time their implication in vasomotion. Under hypoxic condition, opening of TTX-sensitive Na_v_ channels by means of depolarizing agent KCl or enhancement of their basal activity by agonist veratridine induced sustained contraction associated to rhythmic oscillatory contractile activity. We determined that the Na_v_ channels in SMCs contribute to oscillatory activity while those in the sympathetic nerves, which contribute to neurotransmitter release, support sustained contraction.

Vasomotion is a complex phenomenon resulting, at the cellular level, from the fine control of a delicate balance between multiple determinants involving both the membrane compartment and the intracellular compartment. This global mechanism requires membrane oscillators, typically voltage-gated ion channels, ion exchangers and transporters working altogether to follow and control membrane potential, and/or cytosolic oscillators or integrators (e.g., Ca^2+^) to produce oscillatory contractile activity [5]. Membrane potential oscillates in synchronization with both intracellular Ca^2+^ and vasomotor movements [9]. Classically, oscillations follow activation of a depolarizing current [8,23], which in turn opens voltage-gated Ca^2+^ channels enabling Ca^2+^ influx and synchronization of Ca^2+^ waves [24]. Membrane oscillators include K^+^ and Cl^−^ channels and membrane transporters (Na^+^/K^+^-ATPase or TRP channels), yet none of them seems essential [25,26,27].

We show here that Na_v_ channels can play an active role in the vasomotion of the human artery. We unraveled this critical role using the specific Na^+^ channel blocker TTX, which efficiently abolished the spontaneous oscillatory activity of human uterine arteries. We confirmed the presence of Na_v_ channels in these arteries. We recorded a TTX-sensitive I_Na_ in single vascular myocytes, which was in line with previous reports of the presence of TTX-sensitive Na_v_ currents in human SMCs [15,17,18]. Several studies have underlined that I_Na_ current is difficult to record in freshly isolated smooth muscle cells probably due to cellular weakness or membrane damage consecutive to enzymatic dissociation [16,17]. However, and although we recorded I_Na_ in primary culture cells, which could be a limitation in this study, Na_v_ channels α-subunits were evidenced in freshly dissociated SMCs and in arterial tissue. The TTX-sensitive isoforms (Na_v_ 1.2; 1.3; 1.7) could be potentially involved in the TTX-sensitive vasomotion. But, Na_v_ 1.2 and Na_v_1.3 isoforms previously identified in rat arteries [21,22] were not specifically related to oscillatory activity. A TTX-resistant isoform (Na_v_ 1.5) was also detected, but we found no evidence for a significant contribution of a TTX-resistant Na^+^ current in patch-clamp experiments and in vasomotion (oscillations blocked at 10 nM, even in the presence of veratridine). Thus Na_v_ 1.7 could be an interesting candidate for that purpose. Additionally, we observed Na_v_ channels localization at both endothelial and SMCs level in line with previous observations in human cutaneous [28] and mouse cremaster arterioles [29].

Vasomotor oscillations are known to occur at a broad spectrum of frequencies corresponding to “slow” and “fast” waves [30]. Low frequency oscillations are subdivided into groups that depend on vascular endothelium, neurogenic activity and intrinsic smooth muscle activity [31]. Frequency of vasomotion originating from smooth muscle is between 1 and 2 min^−1^ [7], which corresponded to that of the oscillatory activity recorded in our study. We demonstrated that rhythmic activity compatible with vasomotion was induced by activation of Na_v_ channels in favorable hypoxic conditions. Interestingly, the enhanced activity of the Na_v_ channel by veratridine triggered a sustained arterial contraction upon which oscillatory activity was superimposed. In the presence of prazosin, only the oscillatory activity remained. 

The inhibition of sustained contraction with prazosin, an alpha blocker, was in line with the involvement of sympathetic perivascular nerve terminals regulation through release of catecholamine and activation of the α1-adrenergic receptors pathway [21,32]. However, the oscillatory activity was not affected either by prazosin or by guanethidine, an inhibitor of vesicular release of norepinephrine/ATP. This eliminates the hypothesis of a neurogenic origin [33] and, therefore, supports the idea that Na_v_ channels of the SMCs compartment are involved in the genesis and maintenance of vasomotion.

In our study, a low concentration of the specific Na_v_ channels blocker TTX, abolished the oscillatory activity of human uterine arteries. Strikingly, TTX was more efficient than all other blockers tested like nifedipine for Ca^2+^ channels, digitoxin for Na^+^/K^+^-ATPase and KB-R7943 for the Na^+^/Ca^2+^ exchanger. Therefore, Na_v_ channels activity was essential in vasomotion unlike the other components in our experimental conditions. Moreover, we highlighted that Na_v_ channels may control oscillatory contractile activity through two independent mechanisms, both resulting of the Na^+^ influx. The first one involves the depolarization induced by Na^+^ influx leading to activation of voltage-gated Ca^2+^ channels and Ca^2+^ entry. The other one, consecutive to intracellular Na^+^ rise, may involve the increased activities of Na^+^/Ca^2+^ exchanger and subsequent elevation of intracellular Ca^2+^ supporting contractile activity [31,32,34], and also the electrogenic Na^+^/K^+^ ATPase promoting hyperpolarization of cellular membrane when extruding 3 Na^+^ ions against 2 K^+^ entering the cell.

The physiological significance of vasomotion is not clearly defined. It may be important for O_2_ delivery, in particular to improve peripheral flow and tissue oxygenation in conditions of reduced oxygen supply during hypoxia and post-occlusion ischemia [1]. Hypoxia enhances vasomotion and increases frequency of rhythmic oscillations in human and animal arteries in vivo [6,34,35,36] and in vitro [37]. In contrast, increased tissue oxygenation causes depolarization and loss of vasomotion [26]. In our study, we evidenced, in vitro, a contribution of Na_v_ channels to oscillatory activity of human uterine arteries particularly under hypoxic condition. Although KCl-induced depolarization or veratridine was required to trigger oscillatory activity, hypoxic condition largely favored these effects on vasomotion in our experiments.

Hypoxia induces hyperpolarization of SMCs in part through K_ATP_ channels activation and the resulting diffusion of K^+^ ions to the extracellular space in systemic arteries [38,39,40]. In our experiments, hypoxia shifted the contractile response to KCl-induced depolarization, an effect otherwise abolished by the K_ATP_ channel blocker glibenclamide known to prevent hyperpolarization [41]. The contractile response induced by means of low KCl was abolished by TTX in line with involvement of SMCs Na_v_ channels [21,32]. Our observations suggested that in uterine artery SMCs the resting membrane is not negative enough to allow Na_v_ channels opening (likely to be inactivated). Thus, hyperpolarization, such as that induced by hypoxia, renders them available for opening. Overall, our results are in line with an involvement of the oxygen level in vasomotion and highlight Na_v_ channels as an active contributor to that phenomenon. This mechanism may be relevant in human uterine arteries sensitive to hypoxia, particularly during pregnancy and preeclampsia. Coronary arteries, where Na_v_ channels are expressed (data not shown) and I_Na_ current recorded [16], may also be concerned by this regulation in relation with the vasospasms developing in conditions of severe or prolonged hypoxia [42]. In our experiments, Na_v_ channels effectors (veratridine or KCl-induced depolarization) were used to initiate vasomotion of uterine arteries in vitro. Although hypoxic condition favors Na_v_ channels recruitment by voltage change and initiation of vasomotion, hypoxia by itself did not trigger vasomotion.

In our study, all arterial samples were used without any discrimination of the patient’s hormonal status on the incidence and regulation of vasomotricity. This could be a limitation of the study and could explain some variations observed in the contractile responses, in particular for the appearance of spontaneous oscillations. Identification of physiopathological effectors of Na_v_ channels in vivo, including hormonal status, warrants further investigation.

## 4. Materials and Methods 

### 4.1. Tissue Collection, Myocytes Isolation and Cell Culture

Specimens of uterine arteries were obtained from non-pregnant women (aged 40–60 years) undergoing hysterectomy for benign gynecological disorder (*n* = 80). None of the selected patients were on hormone therapy or had a history of cardiovascular disease. Immediately after surgery and the removal of the uterus, the main branch of one uterine artery excised from the parametrium, connective tissues and adjacent myometrium was placed in a physiological saline solution (PSS in mM: 140 NaCl, 5 KCl, 1 MgCl_2_, 0.5 KH_2_PO_4_, 0.5 Na_2_HPO_4_, 2.5 CaCl_2_, 10 HEPES and 10 glucose, pH 7.4) and transported to the laboratory for experimentation within 30 min. 

Smooth muscle cells (SMCs) were isolated enzymatically from media layer of arterial tissue previously dissected [32]. The enzymatic dissociation was achieved by gentle mechanical disruption and the collected cells were used for either RNA extraction or cell culture. Cultured cells were obtained after dilution in smooth muscle cell specific culture medium supplemented with 10% fetal calf serum, and plating in collagen treated petri dishes. The primary cultured cells began to attach and spread out 24–48 h after seeding then they were used for patch-clamp experiments.

### 4.2. Electrophysiological Recordings

Cellular electrophysiological recordings were performed, at room temperature (22–24 °C) on cultured SMCs under the whole-cell patch clamp configuration. Experiments were conducted using an Axopatch 200B amplifier (Axon Instruments, San Jose, CA, USA), interfaced to a Dell microcomputer with a Digidata 1440A Series analog/digital interface (Axon), using pClamp 10 (Axon). Recording pipettes were filled with (in mM): 120 CsCl, 5 MgCl_2_, 11 EGTA, 10 HEPES, 1 CaCl_2_, 5 ATP-Na_2_ and 10 TEA-Cl (pH 7.3 with CsOH). The bath solution contained (in mM): 135 NaCl, 1 CaCl_2_, 1 MgCl_2_, 10 HEPES, 10 glucose and 2 NiCl_2_ (pH 7.4 with CsOH). Our experimental conditions were optimized to record only voltage activated I_Na_: NiCl_2_ (2 mM) in bath solution blocked Cav channels and CsCl (120 mM) instead of KCl in the recording pipette inhibited K^+^ currents. Whole-cell membrane capacitances and series resistances were compensated electronically prior to recording. Voltage errors resulting from the uncompensated series resistance (≤ 8 mV) were not corrected. Experimental data were filtered on-line at 10 kHz prior to digitization and storage. Current/voltage I_Na_ relationships were obtained from I_Na_ current recordings, routinely recorded in response to 150 ms voltage steps to potentials between −70 and +60 mV from a holding potential (HP) of −80 mV. Voltage steps were presented in 10 mV increments at 1 s intervals. 

### 4.3. RNA Extraction, RT-PCR and Real-Time RT-PCR

Total RNA was extracted from either uterine artery or freshly dissociated myocytes using TRIzol^®^ reagent (Invitrogen, Carlsbad, CA, USA) according to the manufacturer’s instructions. DNase-treated (DNase I, Invitrogen) total RNA (1–2 µg) was transcribed into cDNA using Superscript II reverse transcriptase (Invitrogen) and random primer oligonucleotides (Invitrogen). Gene-specific primers for human Na_v_ channel isoforms and GAPDH were designed based on sequences available through PubMed (for primer sequences see Table S1) and were validated by amplification of total RNA from positive controls (human brain, heart and skeletal muscle) and direct sequencing of the PCR product (Genome Express, Meylan, France). End-point PCR reactions were run on 50–100 ng cDNA using a HotStartTaq^®^ polymerase (Qiagen, Courtaboeuf, France). Real-time quantitative PCR was performed in a Light Cycler System (Roche Diagnostics, Meylan, France) in combination with the Absolute QPCR SYBR Green Capillary mix (Abgene, Courtaboeuf, France). Primers selected in Table S1 were of equal efficiency (Eff = 1.9) across the range of template concentrations (1–10 ng cDNA). GAPDH was used to normalize the values for transcript abundance using the Eff^−ΔΔCt^ method with Ct over 35 considered as no expressing.

### 4.4. Immunohistochemistry

Double immunofluorescence labeling for Na_v_ channels and smooth muscle α-actin was carried out on cryostat sections of uterine artery embedded in OCT (Tissue Tek^®^, Sakura, Villeneuve d’Ascq, France) and frozen in isopentane. After fixation in ethanol/acetone (*v*/*v*), tissue sections were first blocked with 5% normal goat serum (Sigma-Aldrich, L’Isle d’Abeau Chesnes, France) in PBS containing 1% BSA, then incubated with the first primary antibody, washed with PBS and incubated with the first fluorescent secondary antibody. Sections were then blocked with 5% normal horse serum (Sigma), incubated with the second primary antibody and finally incubated with the second fluorescent secondary antibody. Nuclei were counterstained with 5 µM TOTO^®^-3 iodide (Molecular Probes, Eugene, OR, USA). Sections were coverslipped with fluorescent mounting medium (DakoCytomation, Carpinteria, CA, USA) and visualized using a BioRad MRC-1024 laser scanning confocal imaging system (Montpellier RIO imaging, MRI platform, Montpellier, France). The primary antibodies used were: a Pan Na_v_ channel antibody at 1/50e dilution for the first labeling step (Alomone Labs Cat# ASC-003, RRID:AB_2040204) and a mouse monoclonal anti-smooth muscle α-actin antibody at 1/1000e dilution (Sigma-Aldrich Cat# A2547, RRID:AB_476701, for the second one. Secondary antibodies were, respectively, Texas Red^®^ Cy-3 anti-rabbit IgG (Vector Laboratories, Berlingame, CA, USA Cat# TI-2000, RRID:AB_2336178) and fluorescein anti-mouse IgG, both diluted 1/100e (Vector Laboratories Cat# FI-2000, RRID:AB_2336176). Negative controls followed the same protocol but without a primary antibody, or with the inclusion of peptide antigens (1 µg of peptide for 1 µg of antibody).

### 4.5. Vascular Reactivity

Experiments were performed on freshly collected human uterine artery and as previously described [43]. Artery specimens were dissected and cut into 2–3 mm-wide rings and the endothelium was removed by rubbing. Rings were placed in conventional organ bath chambers filled with PSS, maintained at 37 °C and continuously bubbled with O_2_. Changes in isometric tension were recorded using an IT1-25 force transducer and an IOX computerized system (EMKA Technologies, Paris, France). After a 60 min equilibration period at a resting tension of 2 g (optimal resting tension previously determined with KCl 80 mM), the contractility and endothelium removing of each arterial segment were assessed after the respective application of 10 µM phenylephrine (PE) and 1 µM acetylcholine (Ach). PE is a direct-acting sympathomimetic amine chemically related to adrenaline and ephedrine. PE is a post-synaptic α_1_-receptor agonist with potent vasoconstrictor property. Acetylcholine (Ach) causes endothelium dependent vasorelaxation. Thus, the absence of functional endothelium was assessed by the lack of response to Ach (1 µM) on the top of PE-induced contraction. After a wash (W) and 20–30 min’ period of stabilization, experimental protocols were started. When necessary, hypoxia was induced by replacing O_2_ (100%) bubbling by N_2_ (100%), an equilibration period was performed for at least 20 min in that condition. Various antagonists, used to block α1-adrenergic receptors (prazosin, 10 µM), Na_v_ channels (TTX, 0.01 or 1 µM), K_ATP_ channels (glibenclamide, 0.1 µM) were incubated for 10 min before challenging the artery with KCl. Dose response curves were cumulative from 5 to 80 mM. Other antagonists, nifedipine (1 µM), digitoxin, (10 µM) and KB-R7943 (10 µM) were used to block respectively Ca_v_, Na^+^/K^+^-ATPase and Na^+^/Ca^2+^ exchanger. Specific protocols are detailed in the figure legends.

### 4.6. Chemical Reagents 

TTX was obtained from Tocris Biosciences (Bristol, UK) and culture medium from PromoCell (Heidelberg, Germany). All other chemicals and compounds were purchased from Sigma-Aldrich (L’Isle d’Abeau Chesnes, France). Nifedipine, glibenclamide were dissolved in DMSO, veratridine in 0.1 N HCl and the remaining compounds in distilled water with further dilutions made from stock solutions with PSS.

### 4.7. Data and Statistical Analysis

All data were expressed as mean ± s.e.m and analyzed using GraphPad Prism (v6.05, RRID:SCR_002798). Dose–response curves were fitted with non-linear regressions and statistical differences were assessed using two-way ANOVA followed by a Bonferroni post-hoc test. EC_50_, E_max_ and E_20_ values, amplitude and period were analyzed using a one-way ANOVA followed by Tukey’s post-hoc test. *p* values lower than 0.05 were considered significant. *n* denotes the number of specimen tissues obtained from different patients and, when the same protocol was run on several rings from the same artery, data were averaged.

## 5. Conclusions

Vasomotion is a complex phenomenon, which involves multifaceted regulatory pathways ranging from neurogenic control to cell synchronization and which certainly depend on vascular beds. This phenomenon of physiological importance is known to be modulated under pathological ischemic conditions. Our study clearly identified the Na_v_ channels as a key new element of vasomotion. Under hypoxic condition, these channels can be involved in cellular synchronization and rhythmicity, fingerprints of vasomotion.

## Figures and Tables

**Table 1:** Summary of EC_50_ values, maximal contraction in gram (E_max_) and the contraction induced by 20 mM KCl expressed as percent of E_max_ (E_20_) for KCl dose–response performed in various conditions: basal O_2_ condition (CTL), hypoxic condition (N_2_) and in the presence of prazosin (Pz), glibenclamide (Glib) or tetrodotoxin (TTX).

Condition		EC_50_ (mM)	E_max_ (g)	E_20_ (%max)
Basal O2	CTL (*n* = 15)	22.0 ± 1.3	6.11 ± 0,6	48.2 ± 5.9
	Pz (*n* = 15)	21.4 ± 1.2	5.14 ± 0.49	47.4 ± 4.5
	Pz-TTX (*n* = 15)	21.5 ± 1.3	5.10 ± 0.39	45.5 ± 4.1
Hypoxia	N_2_ (*n* = 20)	29.1 ± 2 **	4.27 ± 0.73 *	26.5 ± 4.8 **
	N_2_-Glib (*n* = 10)	20.2 ± 1.3 ^§§^	4.63 ± 0.9 *	56.1 ± 4.9 ^§§§^
	N_2_-Pz (*n* = 15)	29.3 ± 1.4 **	4.39 ± 0.76	26.4 ± 3.3 **
	N_2_-Pz-TTX (*n* = 15)	32.7 ± 1.3 ***	3.31 ± 0.61	12.0 ± 1.8 ***^,§,£^

Values are mean ± s.e.m. * *p* < 0.05; ** *p* < 0.01; *** *p* < 0.001 for comparison to CTL values. § *p* < 0.05; §§ *p* < 0.01; §§§ *p* < 0.001 for comparison to N_2_ values and under hypoxia. £ *p* < 0.05 for comparison between N_2_-Pz and N_2_-Pz-TTX.

## Data Availability

Data is contained in the present manuscript or supplemental materials.

## Supplementary Materials

Supplementary materials can be found at https://www.mdpi.com/1422-0067/22/5/2570/s1.

## Abbreviations

Ach	Acetylcholine
INa	Voltage-gated sodium current
Na_v_	Voltage-gated sodium channels
PE	Phenylephrine
SMCs	Smooth Muscle cells
TTX	Tetrodotoxin
