# Supplementary material for: Hypoxic Conditions Promote Rhythmic Contractile Oscillations Mediated by Voltage-Gated Sodium Channels Activation in Human Arteries
Source: Int J Mol Sci. 2021 Mar 4;22(5):2570. doi: 10.3390/ijms22052570 (PMC7961413; doi:10.3390/ijms22052570)
Supplement: Supplementary file 1 [file ijms-22-02570-s001.pdf]

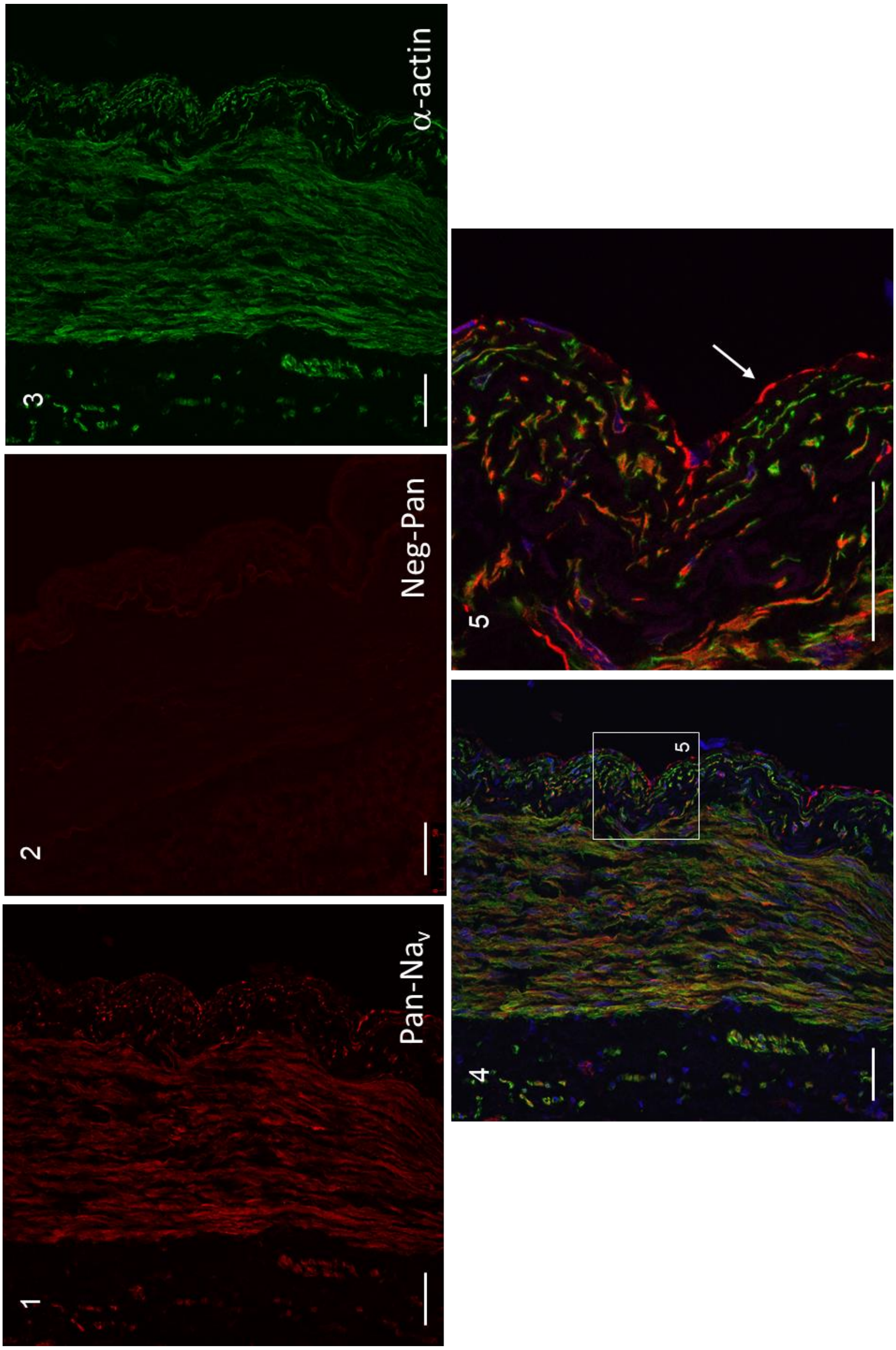

Supplemental figure 1 : Enlarged images presented in figure 2c. Immunolocalization of Na$_v$ channels in uterine artery. Confocal microscopy images show typical labelling of uterine artery section with Pan Na$_v$ channel antibody (**1**) and in the presence of peptide antigen negative control (**2**) (SP19 red fluorescence); α-actin antibody (green fluorescence) (**3**); merged fluorescence with TOTO counterstaining (blue) (**4**); using a x20 objective. (**5**) corresponded to high magnification (60X objective) of the inset in (4) showing endothelial (plain arrow) and smooth muscle (dotted arrow) cells. *Scale bars* - 50 µm.

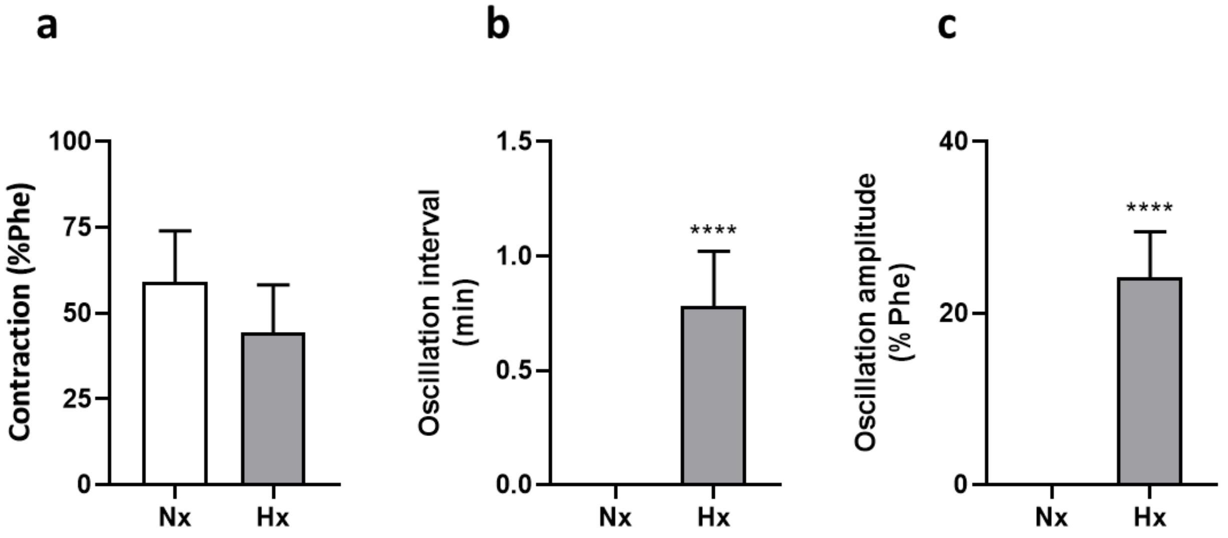

Supplemental figure 2 : Graphs summarizing data for the effect of veratridine under normoxic (Nx, n=15) and hypoxic (Hx, n=5) conditions in the absence of prazosin: contraction (**a**), mean interval between two consecutive oscillations (**b**) and mean amplitude of oscillations (**c**). It is worth to note that under normoxic condition, no oscillation was induced by veratridine. Values are mean ±sem. Statistical analysis was performed using *t*-test. ****p<0.0001.

Isoform	Accession number	Forward primer - 5'-3'	Reverse primer - 5'-3'	Product (pb)
Nav 1.1	AY043484	TAACGAGAGCCGTAGAGAT	CAGGCGATGTAGGAACT	191
Nav 1.2	NM_021007	CTCCAAGACGCAACAG	AGCAGATGTGAGGGTAGAA	296
Nav 1.3	AF225987	AGAAAACTGAAGCCAAGCCA	GAAAAGCTCCAGGTCCCTTC	157
Nav 1.4	NM_000334	AGGGACCTGCTGCTCAGTAA	GAAGTGCTTCTTCAGGCCAC	243
Nav 1.5	NM_000335	GATGTGTTACTGTGTGGG	CTCGGTCTCAGCGATG	321
Nav 1.6	AF225988	AAATCTCTAAACTCAGCTCAAAG	CCAGGTCCCCTGAAAC	280
Nav 1.7	NM_002977	CGTGGACAAACACTTGATGG	CTCCAGGCAAAGGGTTATCA	215
Nav 1.8	NM_006514	ACTCTCCGATGGAAGCAAGA	CTCTCATAGGACGGTGGGAA	236
Nav 1.9	NM_014139	AACTAAAGTCCAGTTAGCAC	CAATCATGCCTGACGC	252
β-1	NM_001037	GTGTATGGGATGACCTTCAAA	GTAGTCGCCAGAGTGG	267
β-2	NM_004588	GATGCCTGGCTACCTCGCCCT	AACCTGAAGCTGGAGCGGTTT	276
β-3	NM_018400	GACTCTGGCCTCTACAC	GCGTCTGACTACCTTGC	260
β-4	NM_174934	ACAGCAGTGACGCATTCAAG	CACATGGCAGGTGTATTTGC	188
GAPDH	NM_014364	AGCCGCATCTTCTTTTG	CCACGACGTACTCAGC	338

Supplemental Table 1: Nucleotide sequences of the specific primers used to amplify Na_v channel α and β mRNA isoforms by real-time RT-PCR. Each set of primers was designed from mRNA sequences with the Genbank accession numbers indicated above, using the primer design software Light Cycler Probe Design (Roche). They generated PCR products of the predicted length in base pairs.
